# Germanium Quantum-Dot Array with Self-Aligned Electrodes for Quantum Electronic Devices

**DOI:** 10.3390/nano11102743

**Published:** 2021-10-16

**Authors:** I-Hsiang Wang, Po-Yu Hong, Kang-Ping Peng, Horng-Chih Lin, Thomas George, Pei-Wen Li

**Affiliations:** Institute of Electronics, National Yang Ming Chiao Tung University, Hsin Chu City 30010, Taiwan; wang.ee06g@nctu.edu.tw (I.-H.W.); allenhong1113@gmail.com (P.-Y.H.); pengkp@hotmail.com (K.-P.P.); hclin@nycu.edu.tw (H.-C.L.); tg4786125@gmail.com (T.G.)

**Keywords:** germanium, quantum dot, self-aligned electrode, scalability

## Abstract

Semiconductor-based quantum registers require scalable quantum-dots (QDs) to be accurately located in close proximity to and independently addressable by external electrodes. Si-based QD qubits have been realized in various lithographically-defined Si/SiGe heterostructures and validated only for milli-Kelvin temperature operation. QD qubits have recently been explored in germanium (Ge) materials systems that are envisaged to operate at higher temperatures, relax lithographic-fabrication requirements, and scale up to large quantum systems. We report the unique scalability and tunability of Ge spherical-shaped QDs that are controllably located, closely coupled between each another, and self-aligned with control electrodes, using a coordinated combination of lithographic patterning and self-assembled growth. The core experimental design is based on the thermal oxidation of poly-SiGe spacer islands located at each sidewall corner or included-angle location of Si_3_N_4_/Si-ridges with specially designed fanout structures. Multiple Ge QDs with good tunability in QD sizes and self-aligned electrodes were controllably achieved. Spherical-shaped Ge QDs are closely coupled to each other via coupling barriers of Si_3_N_4_ spacer layers/c-Si that are electrically tunable via self-aligned poly-Si or polycide electrodes. Our ability to place size-tunable spherical Ge QDs at any desired location, therefore, offers a large parameter space within which to design novel quantum electronic devices.

## 1. Introduction

Since the inception of quantum computing in the early 1980, extensive research on photons [1], ion traps [2], superconducting circuits [3], and semiconductor quantum dots (QDs) [4,5,6] has resulted in spectacular advances in quantum-bit (qubit) technologies potentially facilitating a vast landscape of applications. While impressive achievements have been made using superconducting qubits operating at mK temperatures, semiconductor QD qubits have recently emerged as the subject of intensive research not only for the promise of scalability, but also for their ease of manufacturability using existing very large scale integrated circuits (VLSI) technologies [7,8,9,10,11,12,13,14,15,16]. Pioneering studies on III–V QDs have led to important proofs-of-concept for coherent control of electron-electron and electron-spin interactions [4,5] Group IV semiconductors, Si and Ge, subsequently advanced these concepts to a more practical level due to their promise of relatively straightforward integration with complementary metal-oxide-semiconductor (CMOS) electronics for effective qubit control, read/write, and subsequent signal processing [8,9,10,11,12,13,14,15,16]. Long spin coherence times for the zero-nuclear-spin isotopes of ^28^Si and ^74^Ge, in particular, have made both Si and Ge attractive as the host materials for QD-based spin qubits and affiliated single-electron transistors (SETs) exploiting their charge and spin degrees of freedom [13,14,15].

Semiconductor quantum computers require scalable QD qubits to be accurately located in close proximity to each other and also be independently addressable by external electrodes via tunable coupling. To date, advances in Si-based qubit technology have been demonstrated mostly using lithographically-defined approaches including electrostatically-induced QDs and physically-etched QDs based on two-dimensional electron-gas (2DEG) or hole-gas (2DHG) heterostructures [8,9,10,11,12,13,14,15,16,17] and one-dimensional nanowire structures [18,19]. Among the demonstrated electrostatically-induced QD techniques, overlapping gate architectures [8,20] have offered some flexibility in forming gate-controlled QDs with electrically-tunable coupling between adjacent QDs. At least 2N + 1 control electrodes are required for defining N QDs and creating their confinement barriers. That is, N plunger gates (PGs) are required to set the potential and charge occupation within the QDs in combination with N + 1 intervening barrier gates (BGs) to adjust inter-QD exchange interactions and QD-reservoir coupling. Si/Si_1 − x_Ge_x_ double QDs (DQDs) [10,11,12,13,14,18], triple QDs (TQDs) [21], quadruple QDs (QQDs) [22], and octuple QDs (OQDs) [23] in linear-chain and two-dimensional arrays have been reported for qubit logic gates. But, filling charges within a specific QD among a large overlapping-gate QD array still faces difficult technical challenges such as effective elimination/reduction of qubit cross-talk and quantum-state leakage. The overlapping-gate architecture results in individual gate voltages not only modulating the specific QD potentials or inter-QD coupling that they are designed to control, but also influencing parameters of other, unintentionally-addressed QDs through capacitive cross-talk [24,25]. Also, gate-induced disk- or circular-shaped QDs based on 2DEG/2DHG heterostructures, which have diameters much larger than their thickness, can result in highly anisotropic potential confinement [17,26]. The transverse potential in these structures, in particular, exhibits cylindrical symmetry with a soft-wall profile, resulting in weak confinement and hyperfine energy-level splitting. These effects have resulted in the operation of Si/SiGe qubits only being validated at very low temperatures (≤2 K) [8,16]. Although physically etched QD approaches do indeed increase the freedom for implementing QD devices with diverse spatial orientations and locations [27], a major challenge that remains is the formation of electrical contacts to specific QDs even using the most advanced lithographic techniques available. Besides, hard-wall confined QDs can lead to fixed tunneling rates and fixed exchange interactions between QDs [28], making it extremely difficult to reliably measure very small output current/voltage signals.

For the proper functioning of QD qubits with high fidelity, it is vital to fabricate reliable and scalable QDs with a high degree of control over the QD size, shape, crystallinity, strain, and inter-QD spacing. In particular, the physical dimensions of QDs and their coupling barriers must be sufficiently small at nanometer-scale levels [17]. This last requirement has proven challenging from a fabrication perspective. Controllably producing ultrasmall Si QDs, since this is dictated by the small Bohr radius of 4.9 nm in Si, is difficult using lithographic techniques alone. In contrast, a larger Bohr radius of 24.9 nm in Ge enables easier modification of Ge QD-based device structures, imposing far less stringent demands on lithographic control as compared to Si QD fabrication. Also the co-existence of long electron-spin relaxation times with strong spin-orbit coupling in Ge permits electrically-driven manipulation for fast operation [29]. Encouragingly, the proof-of-principle Ge qubit devices have been experimentally demonstrated using Ge/SiGe planar heterostructures [15,30], Ge hut wires [31], and Ge/Si core/shell nanowires [32], respectively. Progresses in the optimization of Ge hole-based qubit devices based on these material platforms have excited important achievements in terms of large g-factors and spin-orbit interaction energies [33]. Each of these platforms offers specific advantages but also poses challenges, which have been comprehensively elaborated and reviewed in [33]. Thus far, operation of two- and 4-qubit logic gates at mK temperatures has been demonstrated using large Ge QDs (5–70 nm in height and ~100 nm in planar dimensions) with an inter-QD pitch of 150–200 nm based on gate-defined SiGe/Ge/SiGe quantum-wells on Si substrates [15,30,34].

We have reported a CMOS-compatible fabrication approach for the controllable growth of spherical-shaped Ge QDs/SiO_2_ shells within Si-containing layers (SiO_2_, Si_3_N_4_, and Si) in a self-organized manner [35,36,37,38]. Using a coordinated combination of lithographic patterning and self-assembled growth, size-tunable Ge QDs were controllably positioned by successfully exploiting the many peculiar and symbiotic interactions of Si, Ge, and O interstitials [39,40,41]. Our Ge QDs were created using the selective oxidation of poly-Si_1 − x_Ge_x_ lithographically-patterned structures with Si_3_N_4_ in proximity. We have exploited the multi-dimensional parameter spaces of process conditions to grow Ge QDs with a high degree of controllability in the size, morphological shape, chemical purity, crystallinity, and spatial locations [39,40,41,42,43,44,45,46,47]. We have also proven the feasibility of paired DQDs embedded within SiO_2_/Si_3_N_4_ matrices at each sidewall edge of lithographically-patterned Si ridges using spacer technology and thermal oxidation of poly-SiGe [37,38]. The inherent structural simplicity of our self-organized Ge QD/SiO_2_ shell heterostructures perfectly enables the experimental realization of Ge-QD single-hole transistors (SHTs) [42,43,44]. Well-resolved tunneling current spectroscopy and superior charge stabilities measured at T = 77–150 K [43,44], suggests that our Ge-QD SHTs are effective charge sensors.

**Figure 1:**
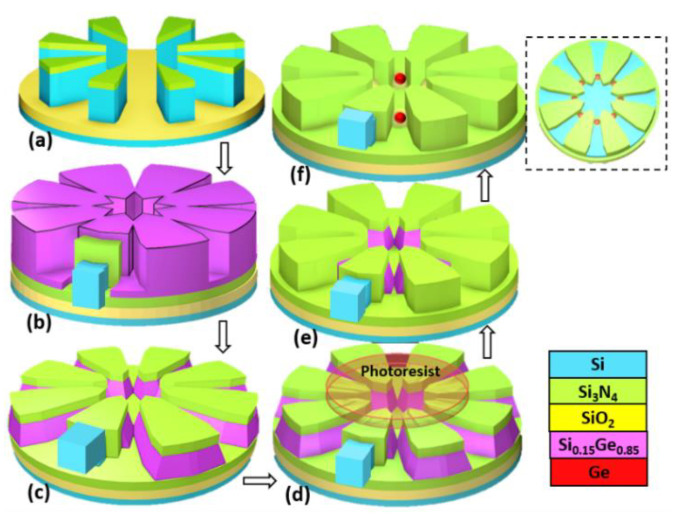
Process flow diagrams showing the fabrication of multiple QDs embedded within SiO_2_/Si_3_N_4_ matrices via the thermal oxidation of SiGe spacer islands at designated included-angle locations of Si_3_N_4_/c-Si ridges. (**a**) Lithographically patterned Si_3_N_4_/c-Si fanout ridges on top of an SOI substrate. (**b**) Next, sequential deposition of Si_3_N_4_ and poly-Si_0.85_Ge_0.15_ layers conformally encapsulates the Si_3_N_4_/c-Si ridges. (**c**) Symmetrical spacer stripes of poly-Si_0.85_Ge_0.15_ are subsequently fabricated at each sidewall of the Si_3_N_4_/c-Si ridges by a direct etch back process. (**d**) Lithographic-patterning shadowing the central regions of the designed fanout ridges in combination with (**e**) etching processes are conducted to define the lengths of the poly-Si_0.85_Ge_0.15_ spacer islands. (**f**) Next, spherical Ge QDs are formed at each included-angle location of the nano-patterned Si_3_N_4_/c-Si ridges sidewall by thermal oxidation. The inset is the plan-view sketch showing the simultaneous formation of multiple Ge QDs at each included-angle locations of Si_3_N_4_/c-Si ridges by design.

In this paper, we advance the self-aligned fabrication of ordered arrays of Ge QDs closely coupled with each other via Si_3_N_4_ spacer layers/c-Si ridges that serve as inter-QD coupling barriers. The core experimental design is based on the thermal oxidation of poly-SiGe spacer islands located at each included-angle location of specially designed Si_3_N_4_/Si-ridges (Figure 1). By tailoring the specially designed fanout structures, Ge multiple QDs with good tunability in QD sizes were controllably generated at each included-angle location of Asterisk-shaped Si_3_N_4_/Si ridges.

## 2. Experimental Methods and Procedures

**Figure 2:**
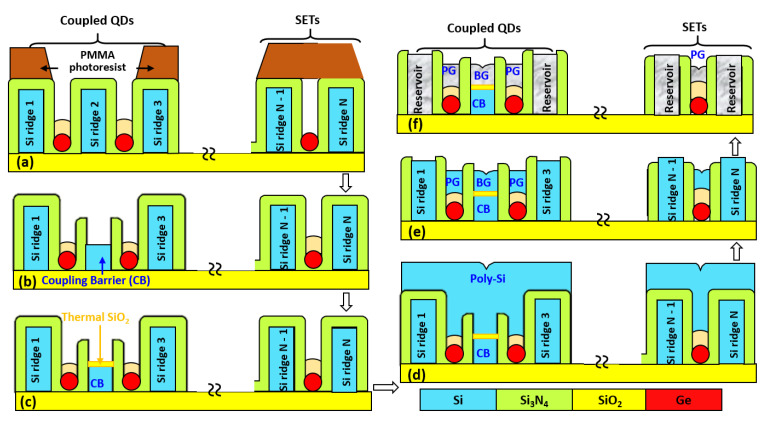
Process flow diagrams showing the fabrication of self-aligned PGs, BGs, and source/drain reservoirs. (**a**) EBL opening of the selected c-Si ridges (ridge #2) that would serve as CBs; (**b**) the removal of the top Si_3_N_4_ and the top portion of the selected c-Si ridges (ridge #2) using CHF_3_ plasma and SF_6_/C_4_F_8_ plasma, respectively, forming CBs; (**c**) the growth of a thin thermal SiO_2_ over the CBs; (**d**) deposition of poly-Si overlayers; (**e**) direct etch back of poly-Si overlayers and top Si_3_N_4_ over the outmost c-Si ridges (for instance, ridges #1, #3, #N - 1, #N), forming PGs over the Ge QDs and BGs over CBs via SiO_2_ layers; (**f**) silicidation of the outmost c-Si ridges, PGs, and BGs, forming polycide reservoirs, PG, and BG, respectively.

The experimental procedure for the fabrication of self-organized Ge multiple QDs with coupling barriers of Si_3_N_4_/c-Si ridges and self-aligned Si electrodes (BGs, PGs, and reservoirs) is described in Figure 1 and Figure 2, respectively. Starting with a silicon-on-insulator (SOI) substrate comprising a 100 nm-thick single-crystalline Si (c-Si) layer and a 400 nm-thick buried SiO_2_ layer on top of Si substrate, a 25 nm-thick Si_3_N_4_ layer was deposited using low-pressure chemical vapor deposition (LPCVD) as the hard-mask layer for the subsequent processes of plasma etching and thermal oxidation. Specially designed Asterisk-shaped Si ridges were subsequently produced using a combination of electron-beam lithographic (EBL) patterning and SF_6_/C_4_F_8_ plasma etching (Figure 1a). Next, bi-layers of 10 nm-thick Si_3_N_4_ and 25–30 nm-thick poly-Si_0.85_Ge_0.15_ were sequentially deposited using LPCVD (Figure 1b) for conformal encapsulation over the Si ridges. Following a direct etch-back process using SF_6_/C_4_F_8_ plasma (Figure 1c), spacer stripes of poly-Si_0.85_Ge_0.15_ with width/height of 20–30/10–30 nm were symmetrically produced at each sidewall of the Si_3_N_4_/c-Si ridges by adjusting the etch-back process time. A second EBL (Figure 1d) in combination with SF_6_/C_4_F_8_ plasma etching (Figure 1e) was conducted for shadowing the central regions of the Asterisk-shaped Si_3_N_4_/Si ridges, respectively. In this way we defined the lengths of the poly-Si_0.85_Ge_0.15_ spacer islands at each included angle location of the Si_3_N_4_/Si ridges (Figure 1e). Subsequently, thermal oxidation at 900 °C for 25–40 min in an H_2_O ambient was performed to convert these poly-Si_0.85_Ge_0.15_ spacer islands to Ge QDs with cladding oxide layers (Figure 1f) at designated locations by the ridges.

Following the formation of cladding oxide/Ge QDs at the included-angle locations of the fanout-ridge structures, EBL process opened the selected regions of Ge QDs and coupling barriers (CBs) of Si_3_N_4_ spacers/c-Si ridges (that is, shadowing the outmost c-Si ridges with photoresists) as shown in Figure 2a. Next, the top Si_3_N_4_ and the top portion (~50 nm-thick) of c-Si ridges (Figure 2b) were sequentially removed using CHF_3_ plasma and SF_6_/C_4_F_8_ plasma, respectively. A subsequent thermal oxidation process grew a 5 nm-thick SiO_2_ layer on top of the selected c-Si ridges (Figure 2c). Next, combined processes of deposition (Figure 2d) and direct etch-back (Figure 2e) of 100 nm-thick poly-Si layers simultaneously form plunger gates (PGs) on top of the capping SiO_2_/Ge QDs and barrier gates (BGs) over the 5 nm-thick SiO_2_/c-Si ridges in a self-aligned approach. Finally, the poly-Si plunger gates, barrier gates, and the outmost c-Si ridges (serving as reservoirs) could be converted to metallic electrodes of NiSi by using the self-aligned silicidation processes (Figure 2f).

In this work, the critical lithographic patterning of Si ridges and SiGe spacer islands was conducted using Raith VOYAGER electron-beam lithography system (Raith GmbH, Dortmund, Germany) and Oxford DSiE plasma etcher (Oxford Instruments plc, Abingdon, UK). Thin specimens for scanning transmission electron microscopy (STEM) observation were prepared by ion-beam milling in a dual-beam (focused ion beam and electron beam) TESCAN GAIA3 (TESCAN, Brno, Czech Republic) using in-situ liftout techniques in order to reduce carbon contamination levels during sample preparation. Energy dispersive x-ray spectroscopy (EDS) analyses were carried out in a FEI Titan G^2^ 80-200 ChemiSTEM (FEI Technologies Inc., Salem, OR, USA), equipped with a Cs probe corrector in combination with an in-column Super-X EDS (Bruker Corporation, Billerica, MA, USA) with four windowless silicon-drift detectors (4 × 30 mm^2^) and operated at 200 kV, leading to a spatial resolution of 7 Å. All STEM imaging and EDS analyses were performed by using a high-angle annular dark-field (HAADF) detector (E.A. Fischione Instruments, Inc., Export, PA, USA) with convergence semi-angles of 8.24 mrad for the inner acceptance angle and ~143.6 mrad for the outer acceptance angle at spot size 9. The characteristic X-ray fluorescence energy lines for Germanium, Silicon, Nitrogen, and Oxygen are Ge-Kα: 9.871 keV, Si-Kα: 1.74 keV, N-Kα: 0.392 keV, and O-Kα: 0.525 keV, respectively. SEM examinations were conducted using a Hitachi S-4700I field-emission scanning-electron microscope (Hitachi High-Technologies Corp., Tokyo, Japan) at an acceleration voltage of 15 kV with a resolution of 1.5 nm. Synchrotron X-ray diffraction (XRD) measurement was performed in the BL07 beamlines of National Synchrotron Radiation Research Center (NSRRC), Hsinchu, Taiwan. Incident X-ray (wavelength 0.6888 Å, 18 keV) was generated from a superconducting undulator and, consequently, X-ray with ultra-high flux could be obtained. When we precisely controlled two angles for single crystal diffractions in the double crystal monochromator, energy resolution of X-ray achieved 1.5 × 10^−4^ ΔE/E). An imaging plate detector (Mar345, made by marXperts GmbH, Norderstedt, Germany) was used to collect Laue rings, and a CeO_2_ powder standard was used to calibrate incident X-ray energy, sample-to-detector distance, and title/rotation of a detector. Finally, diffraction patterns were obtained as integrating Laue ring by GSAS II package.

Temperature-dependent current-voltage (I-V) measurements were conducted in a Lakeshore TTP-6 liquid-nitrogen cooled vacuum-sealed probe station (Lake Shore Cryotronics, Inc., Westerville, OH, USA) using the semiconductor device analyzer Agilent B1500A equipped with B1517A high-resolution source monitor unit/atto sense and switch unit (Keysight Technologies, Santa Rosa, CA, USA), improving the low-current measurement resolution to femtoampere range. Kevin triaxial cables were used to connect the B1500A to wafer probers for these cables producing less electrical noise, leakage, and electromotive force than doing standard triaxial cables. The set-up parameters of B1500A for the current characterization is summarized as follows: hold time: 1 s, delay time: 10 ms, and integration time: 0.6 s, providing a null current of <1 fA at 77 K. The differential conductance, *G*_D_ ≡ ∂*I*_D_/∂*V*_D_, was obtained by numerical smoothing measured *I*_D_−*V*_D_ data using a simplified least squares procedure and then making differentiation.

## 3. Results

### 3.1. Formation of Self-Assembled, Closely-Coupled Ge QDs Arrays

**Figure 3:**
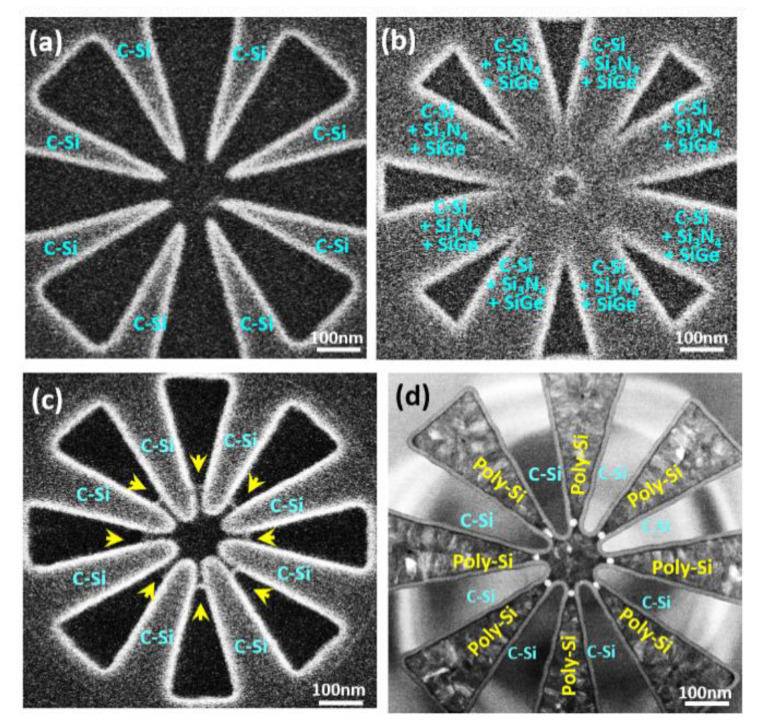
Plan-view SEM/TEM micrographs showing key process steps for the fabrication of closely-coupled Ge octuple QDs located at the included-angle locations of c-Si ridges with specially designed, asterisk-shaped fanout structures. SEM/TEM observations of (**a**) Lithographically patterned c-Si fanout ridges formed on top of SOI substrates. (**b**) Sequential deposition of Si_3_N_4_ and poly-Si_0.85_Ge_0.15_ layers conformally encapsulating the c-Si ridges. (**c**) Symmetrical spacer layers of poly-Si_0.85_Ge_0.15_ fabricated at each sidewall of the Si_3_N_4/_poly-Si ridges by a direct etch back process using SF_6_/C_4_F_8_ plasma. Poly-Si_0.85_Ge_0.15_ spacer islands (highlighted by yellow arrows) were produced at the central regions by using lithographic-patterning and plasma-etching processes. (**d**) Formation of closely-coupled Ge QDs with diameter of 15 nm at each included-angle location of the c-Si fanout ridges following thermal oxidation. Poly-Si layers were then deposited forming plunger gates and barrier gates.

Our fanout fabrication process promises to ultimately achieve the controllability necessary for simultaneously forming closely-coupled multiple Ge QDs (Figure 1) with self-aligned barrier gates and plunger gates as shown in Figure 2 via adjustable coupling barriers of Si_3_N_4_/c-Si ridges and capping SiO_2_, respectively. These unique heterostructures were obtained by the thermal oxidation of poly-SiGe “spacer islands” located at each included-angle location of the specially designed, fanout-shaped Si ridges. Figure 3 shows the plan-view SEM/STEM micrographs of the key process steps for the fabrication of closely-coupled, octuple Ge QDs with diameters of 15 nm at each included-angle location of the c-Si fanout ridges.

### 3.2. Arrays of Ge QDs with Scalable Numbers and Tunable Diameters

**Figure 4:**
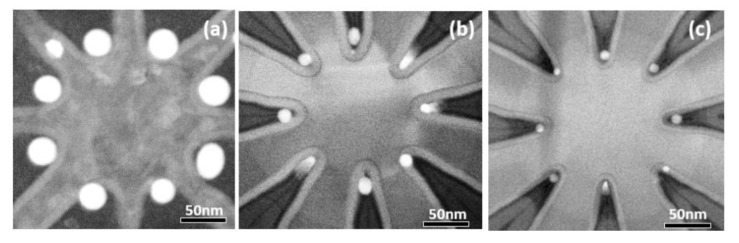
Plan-view STEM observations of (**a**) 30 nm; (**b**) 15 nm; and (**c**) 8 nm Ge octuple QDs fabricated at the sidewall corner of each included angle location for the Si_3_N_4_/asterisk-shaped c-Si ridges showing exquisite control of the number and sizes of the QDs.

The overall number of Ge QDs in the configuration is essentially determined by the fanout number of the c-Si ridges via positioning a single Ge QD at each included-angle location. Figure 4 shows Ge OQDs configurations created by using Asterisk-shaped Si ridge geometry with eight fanouts. Process-controlled tunability of the Ge QD diameter is achieved by adjusting the overall Ge content of the poly-Si_0.85_Ge_0.15_ spacer island. The width and height are varied by controlling the process times for deposition and etch back, respectively, of the poly-SiGe spacer layers. Finally, the exposure dose of EBL for defining the poly-SiGe spacer islands determines their length and hence the overall Ge content. It is clearly seen from the plan-view STEM micrographs in Figure 4 that Ge OQDs with diameters of 30, 15, and 8 nm, respectively, appear at each included-angle location of the Si_3_N_4_/Si ridges following thermal oxidation (at 900 °C for 25 min) of poly-Si_0.85_Ge_0.15_ islands with widths/heights/lengths of 30/45/60 nm, 25/40/40 nm, and 20/20/30 nm.

### 3.3. Arrays of Ge QDs with Self-Aligned Electrodes

**Figure 5:**
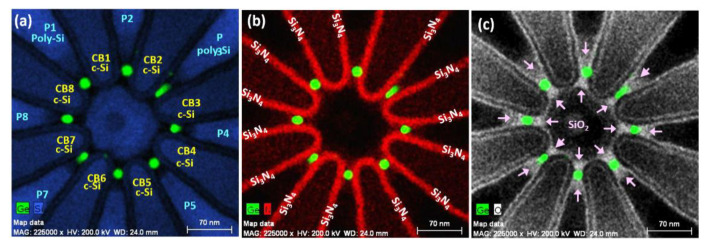
Plan-view EDS maps of elemental (**a**) silicon (Si—blue), germanium (Ge—green); (**b**) nitrogen (N—red), Ge; and (**c**) oxygen (O—white), Ge of octuple Ge QDs fabricated at each included-angle location of asterisk-shaped Si ridges with Si_3_N_4_ overlayers.

The engineering advantages of our Ge QD fabrication approaches not only include process-controlled placement of size-tunable Ge QDs at designated locations, but also offer a feasible integration scheme for forming self-aligned electrodes. That is, the potentials of Ge QDs and inter-QD coupling barriers of Si_3_N_4_/c-Si ridges are electrically adjustable by controlling the poly-Si (or polycide) plunger-gates and barrier-gates, respectively, through thermally-grown SiO_2_ layers. The EDS maps of elemental Si, Ge, nitrogen (N), and oxygen (O) micrographs in Figure 5 show that the inter-QD spacings of 30–50 nm are essentially determined by the widths of lithographically-patterned c-Si ridges in combination with the sidewall thicknesses of the Si_3_N_4_ overlayers. That is, the Si_3_N_4_ spacer layers and c-Si ridges directly define inter-QD coupling barriers (Figure 5a,b). Concurrent with the formation of Ge QDs, their cladding layers of SiO_2_ were also generated from the selective oxidation of the Si content of poly-SiGe spacer islands (Figure 5c).

Following the formation of Ge QDs and their cladding layers of thermally-grown SiO_2_, combined processes of EBL, etch-back (Si_3_N_4_ and c-Si), and thermal oxidation were sequentially conducted on the selected c-Si ridges that would serve as coupling barriers (Figure 2a,b). Subsequent deposition and etch back processes of poly-Si layers produce self-aligned poly-Si barrier gates on top of SiO_2_/coupling barriers of c-Si ridges and self-aligned poly-Si plunger gates over the cladding SiO_2_/Ge QDs. Poly-Si barrier gates electrically adjust effective barrier width of the spacer Si_3_N_4_ layers and barrier height of c-Si ridges and thereby modulate inter-QD charge-charge exchange interactions.

## 4. Discussion

Vital requirements on semiconductor QDs for functional quantum electronic devices include (1) the control over crystallinity and crystal orientations of QDs, (2) the adjustability of the QD sizes and morphological shapes with controllable positions by design, (3) good interface properties of QDs/confinement barriers, and (4) strain engineering in the QDs for valley splitting.

Our previous reports have already conducted extensive STEM-EDS and electron energy loss spectroscopy (EELS) line scan/map examinations, confirming the high chemical purity of Ge QDs (no alloyed Si or Oxygen present within the QD) [45,46]. Clear lattice fringes observed in high-resolution TEM micrographs and sharp diffraction spots observed in the selected area electron diffraction (SAED) patterns are testament to the good crystallinity of our Ge QDs [43,44,45,46]. Raman spectroscopy [46,47] and photoluminescence (PL) [47,48] measurements also confirm the high degree of crystallinity within the Ge QDs in terms of sharp Raman phonon lines and temperature-insensitive PL peaks, respectively.

Our systematic Raman measurements in combination with TEM/SAED examinations also reveal an important observation that the local environments of SiO_2_ and Si_3_N_4_ have a significant influence on the sign of the strain, tensile or compressive, which is imposed on the Ge QDs [46,47] That is, compressive and tensile strains can be generated in our Ge QDs depending on whether the Ge QD is embedded within Si_3_N_4_ or SiO_2_ layers. Measured Grüneisen parameters from temperature-dependent Raman frequencies suggest significant anharmonicity for small Ge QDs with possible distortions of the diamond cubic lattice, which have been confirmed by their lattice spacings through the transmission electron diffraction patterns. We have also observed that quantum phonon confinement effect sets in when the Ge QD size is smaller than 40 nm [47,48,49]. Therefore, the valley degeneracy in our Ge QDs could be split by tailoring the local environmental materials of SiO_2_ or Si_3_N_4_ in combination with adjusting the QD sizes by design.

From device fabrication perspectives, making source/drain reservoirs and creating tunable tunnel barriers/coupling barriers to specific, small self-assembled QDs are very challenging, in general, requiring very precise overlay alignment by means of advanced lithography. In this work, we advance the fabrication of ordered arrays of Ge QDs with self-organized tunnel barriers/coupling barriers and self-aligned electrodes. The appeals of our proposed Ge QD approach lie in the engineering advantages of controllably positioning size-tunable spherical Ge QDs with a high degree of crystallinity at desired spatial locations and thereby offering a large parameter space within which to design novel quantum electronic devices.

### 4.1. Self-Organized, Crystalline Ge QD/SiO_2_-Shell with Si_3_N_4_/c-Si Coupling-Barrier Layers

Our self-organized Ge QD arrays with tunable QD sizes, scalable numbers of QDs, and their controllable placement at designated locations were constructed on specially-designed Si-ridge structures encapsulated with conformal overlayers of Si_3_N_4_. The Si_3_N_4_ overlayers are pivotal for shaping and positioning the Ge QDs. It is also important to note that the Si_3_N_4_ spacer layers together with the c-Si ridges directly define coupling barriers between adjacent QDs.

**Figure 6:**
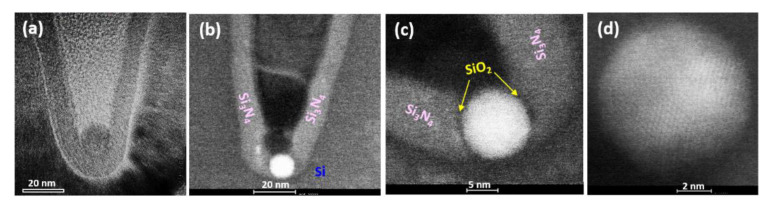
Plan-view TEM/STEM observations of the process evolution of Ge QDs formed at the included-angle locations of the asterisk-shaped Si_3_N_4_/Si ridges undergoing thermal oxidation at 900 °C for (**a**) 30 min and (**b**) 40 min. High-resolution STEM observation of (**c**) Ge QD with a conformal SiO_2_ shell penetrating the spacer layer of Si_3_N_4_. Clear lattice fringes shown in (**d**) high-resolution STEM micrograph are testament to the good crystallinity of the Ge QDs embedded within the Si_3_N_4_ layers.

The fabrication process for generating our self-organized Ge QD/SiO_2_ shell within Si_3_N_4_/Si layers is briefly described as follows. Thermal oxidation (850–900 °C) of Si_1 − x_Ge_x_ results in the preferential oxidation of its Si content, converting it to SiO_2_, due to the large difference in the heats of formation of SiO_2_ (−200 kcal/mol) and GeO_2_ (−130 kcal/mol) [50]. The resultant host matrices of SiO_2_ therefore contain a combination of pure Ge nanocrystals and residual Ge interstitials. Among our first interesting and counter-intuitive findings was the fact that the Ge nanocrystals and their associated Ge interstitial clouds catalyze the local decomposition and oxidation of the proximal Si_3_N_4_ layer [40,51]. This decomposition process releases Si interstitials [39,40,41] that in turn, promote the Ostwald ripening and migration of the Ge nanocrystals through their surrounding SiO_2_ matrix in the direction of the Si interstitial concentration gradient towards the Si_3_N_4_ layer (Figure 6a). Concurrent with their migration, the Ge nanocrystals grow in size by Ostwald Ripening culminating in complete coalescence, ultimately resulting in the formation of spherical Ge QDs embedded within Si_3_N_4_ layers (Figure 6b,c) with a high degree of crystallinity (Figure 6d).

The unique penetration of Ge QDs through the surrounding SiO_2_ matrix (Figure 6a) and proximal Si_3_N_4_ layers (Figure 6b) is activated by dynamic SiO_2_ destruction-construction mechanisms near the QD surface [39,40,41]. As the Ge QD ultimately penetrates the entire Si_3_N_4_ layer, a thin conformal SiO_2_ shell is formed separating the Ge QD and the surrounding Si_3_N_4_ (Figure 6c). The SiO_2_-shell thickness of 1–2 nm between the penetrating Ge QD and the Si_3_N_4_ is essentially determined by a dynamic equilibrium that exists between the local concentrations of O interstitials near the Ge QD/Si_3_N_4_ interfaces supplied by the external oxygen ambient, and combined with the concentration of Si interstitials released from the locally decomposing Si_3_N_4_ layer [40,41].

### 4.2. Ge QD Mediated Densification of Proximal Si_3_N_4_ Barriers

**Figure 7:**
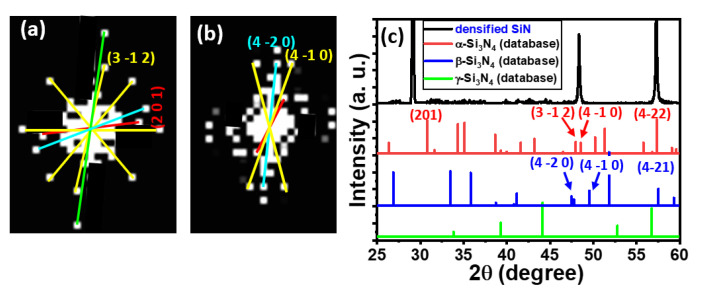
(**a**,**b**) SAED patterns; and (**c**) XRD spectra acquired for the densified, polycrystalline Si_3_N_4_ generated in proximity to the Ge QDs. The appearance of clear electron diffraction spots and XRD peaks is convincing evidence for the phase transition of the Si_3_N_4_ from the amorphous to the densified polycrystalline state.

The next interesting finding was that the penetrating Ge QDs also remarkably mediate the local densification of the nominally amorphous Si_3_N_4_ spacer layers (Figure 7a) via a phase transition from amorphous to the nanocrystalline state, as evidenced by clear diffraction spots in the SAED patterns (Figure 7b) and sharp peaks in the XRD spectra (Figure 7c). The observed peaks at 2θ = 29.12, 48.37, and 57.31° correspond to the crystal planes of (2 0 1), (3 −1 2) or (4 −1 0), and (4 −2 2), respectively, of crystalline Si_3_N_4_ in the α-phase state of a trigonal crystal structure. The derived classification of crystal planes from the XRD spectra and the corresponding diffraction spots identified within the SAED are in good agreement. This densification of Si_3_N_4_ also leads to the reduction in the concentration of hydrogen induced traps and thereby a significant improvement in the trap-assisted tunneling or hopping [49,52]. Low interface trap density (Dit) of ~2–3 × 10^11^ cm^2^ eV^−1^ was measured on the Ge QD/Si_3_N_4_ structures [53], and estimated number of interface traps for a 10 nm Ge QDs/Si_3_N_4_ structure is approximate unity.

### 4.3. Process-Controlled Placement of Spherical-Shaped Ge QDs at Designated Spatial Locations

Placement of our Ge QDs by design is facilitated via controlled heterogeneous nucleation and growth within lithographically patterned structures. Pattern-dependent oxidation and Ostwald ripening-based migration behavior offer additional mechanisms for controlling the QD locations. Our extensive experimental observations show that segregated Ge nuclei tend to form at the sidewall edges and near the included-angle locations of the Si_3_N_4_/c-Si ridges of asterisk-shaped configurations. These locations are also where large geometric curvatures and higher film stress occur. The preferential formation of Ge QDs at the highly stressed ridge sidewall edges and their included-angle locations could also be due to the higher density of defects at these locations and the stress relief provided by the growing Ge QD [47,54]. The insertion of a S_i3_N_4_ overlayer with controllable thickness between the poly-SiGe spacer island and the c-Si ridge provides the tunability necessary for precise Ge QD location. Not only can we direct the Ge QDs migrating towards the designated spatial locations by creating a gradient in the concentration of released Si interstitials in order to activate the dynamic SiO_2_ destruction-construction mechanisms ahead of the migrating Ge QD surface, but also the sacrificial consumption of the Si_3_N_4_ layer prevents the c-Si ridges themselves from being consumed up during the selective oxidation process. In this way, the inter-QD coupling barriers are directly defined by the process-controllable thicknesses of the Si_3_N_4_ spacer layers and widths of lithographically-defined c-Si ridges.

### 4.4. Process-Controlled Size Tunability of Ge Spherical QDs for Operation in Few-Charge Regimes

As mentioned previously, our Ge QDs are created by using the selective oxidation of SiGe spacer islands resulting in progressive segregation, condensation, and Ostwald ripening of Ge interstitials, ultimately producing spherical-shaped Ge QDs. Thus, the Ge QD diameters are, by definition, smaller than the geometric sizes of the initial poly-SiGe spacer islands. The widths and heights of the initial SiGe spacer islands are well controlled to nanometer-scale precision by adjusting the process times for deposition and etch-back, while their lengths are essentially determined by lithographic patterning. Hence, our “hybrid patterning/self-assembly” Ge QD fabrication approach allows a higher degree of controllability for producing ultrafine QDs as compared to processes using lithography alone.

Our Ostwald ripened Ge QD assumes a perfectly spherical shape as predicted by Stekolnikov and Bechstedt [55], since their unique, solid-state migration behavior mechanically decouples the QD from its surrounding matrices of SiO_2_, Si_3_N_4_ or Si. In contrast to the highly orientation-dependent energy subbands with anisotropic, hyperfine energy-level splitting for the gate-defined Ge QDs created from heterostructures of Ge/SiGe quantum-wells or nanowires [15,30,33,34], the spherical shape of our Ge QDs is desirable for quantum-electronic devices. This is because a spherical QD has a three-dimensional, radially symmetric electrostatic potential, giving rise to atomic-like discrete orbitals [26]. Similar to the case of atomic orbitals (1s, 2s, 2p, 3s, 3p, …), these orbitals are also filled sequentially with large addition energies for complete filling of shells with 2, 10, 18 electrons [26]. In particular, when the sizes of spherical Ge QDs are comparable to the Bohr’s radius (~24.9 nm) or the de Broglie wavelength and smaller, the well separated energy levels in combination with large addition energies allow the QD devices to operate in the few-charge regime. The special interest in few- and even single-charge operating regimes arises from the fact that intra-QD electron-electron interaction dominates. These operating regimes make it possible to form QD-based qubits and QD-based SETs by exploiting the electron filling and spin degrees of freedom while suppressing cotunneling and thermal noise/fluctuation effects because the spin- or charge-states are energetically well-defined and separated from other states.

### 4.5. Ge QD Array for Qubits and Charge Readout Sensors

For the case of the much smaller Si-based QDs, in addition to the challenges associated with the fabrication of closely coupled QDs, another major challenge for the practical implementation of Si QD-based qubits is the reliable measurement of quantum states within these QDs that are susceptible to environmental temperatures and defects. High-precision charge and differential current/voltage sensing devices and associated techniques are definitely required for measuring very small output current/voltage signals (on the order of sub-nA and sub-mV, respectively) for QD qubits.

An SET or SHT, comprising a single QD capacitively coupled to source/drain reservoirs and plunger-gates through confinement barriers, is the ultimate embodiment for electronic devices controlling itinerant current with single charge precision based on Coulomb blockade effects. Their extremely high sensitivity to the charge number makes QD-SETs (or SHTs) excellent readout devices for charge- and spin-qubits. Therefore, having QD-SETs (or SHTs) favorably arranged in close proximity to the QD qubits allows us to sense minute variations of local potentials induced by charge movement in between QDs.

**Figure 8:**
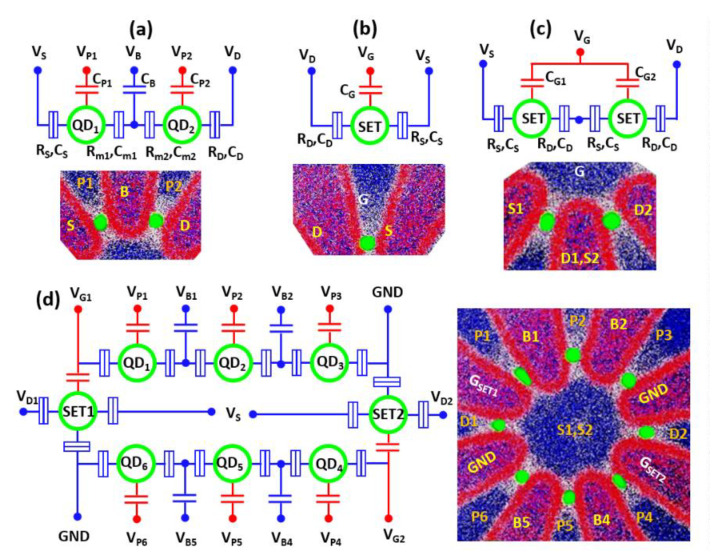
Possible configurations and layouts of Ge QD quantum electronic devices. (**a**) coupled DQDs as a qubit; (**b**) a QD-SET; (**c**) a SET logic inverter comprising two QD-SETs connected in series; and (**d**) a chain of QD-qubits integrated with two charge sensors of SETs.

Our proposed self-organized Ge QDs arrays with self-aligned electrodes offer configurable flexibility in constructing QD-qubits or QD-SETs, depending on the c-Si ridges serving as inter-QD coupling barriers or simply acting as reservoirs. Using our proposed fabrication processes for self-aligned external electrodes of plunger gates, barrier gates, and reservoirs (Figure 2), each QD within the array is individually addressable by four self-aligned electrodes, that is, two poly-Si (or polycide) barrier gates (or one barrier gate and one reservoir), one poly-Si (or polycide) plunger gate, and a common poly-Si (or polycide) layer located in the center of the array. The barrier gates capacitively adjust inter-QD interactions within the coupling barriers of Si_3_N_4_ spacer/c-Si ridge through a thin SiO_2_ layer, whereas the QD potential itself could be independently adjusted by means of the plunger gates or by the common electrode in the center of the array coupled by the newly-grown cladding layers of SiO_2_. For our demonstrated OQD array arrangements, possible QD device configurations are proposed in Figure 8. Figure 8a is a suggested coupled QDs configuration for a qubit including DQDs, one barrier gate over the coupling barrier of Si_3_N_4_ spacer/c-Si fanout-ridge, two plunger gates over the cladding oxide/Ge QDs, and two source/drain reservoirs. A QD-SET configuration comprising a single Ge QD, Si_3_N_4_ spacer layers as tunnel barriers, c-Si ridges as source/drain reservoirs, and a poly-Si plunger gate is proposed in Figure 8b. Another possible configuration for a SET-inverter shown in Figure 8c comprises two QD-SETs connected in series by sharing the same c-Si ridge as reservoirs and modulated by the common poly-Si plunger gate at the center of the array. Figure 8d is a proposed configuration of six QDs in a circular-ring arrangement and closely-integrated with two SETs located at the left and right terminals for proximal charge-sensing.

### 4.6. Proof-of-Principle Ge-QD Single-Hole Transistors Operation

**Figure 9:**
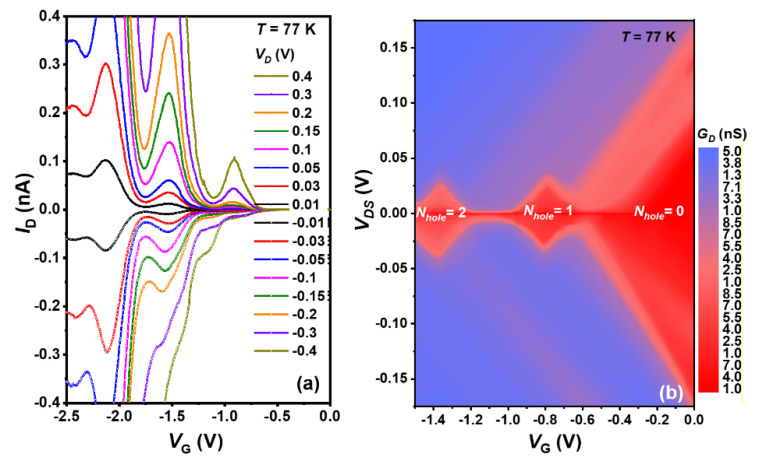
(**a**) I_D_-V_D_-V_G_ curves and (**b**) Coulomb stability diagram of Ge-QD SHTs measured at T = 77 K.

Based on our proposed self-organized heterostructures (Figure 8b) of c-Si (source)/Si_3_N_4_/Ge-QD/Si_3_N_4_/c-Si (drain) in combination with self-aligned poly-Si electrodes (plunger gates), we have fabricated and demonstrated Ge-QD SHTs operation at 77 K. Figure 9 shows experimental characteristics of I_D_-V_D_-V_G_ curves (Figure 9a) and Coulomb stability diagram (Figure 9b) of *G*_D_ contour plot measured at temperature of 77 K. Clear oscillatory current behaviors and well-sealed Coulomb diamonds are testament to the proof-of-principle Ge-QD electronic devices operation. Each oscillatory current peak corresponds to a change of one additional hole within the Ge QDs as a result of strong Coulomb blockade effect. Each node between Coulomb diamonds represents one additional hole tunneling through one-particle energy levels or overcoming particle Coulomb interactions. Estimated single addition energy for holes through the Ge QDs are larger than 25 meV from the slopes and voltage periodicity of the corresponds diamonds in Figure 9b.

## 5. Conclusions

An ingenious combination of lithography and self-assembled growth has allowed us to have accurate control over the placement, shapes and sizes of our “designer” Ge QDs. One novel implementation is the fabrication of closely coupled Ge QDs at designated included-angle locations of specially designed c-Si fanout-ridges providing a common platform for creating diverse QD-based quantum-electronic devices. The appeal of our Ge QD fabrication approach lies in the engineering advantages of positioning the desired number of size-tunable spherical Ge QDs at designated locations. These size-tunable Ge QDs not only share an inter-QD coupling barrier of Si_3_N_4_ spacer layers/c-Si ridges in a self-organized manner, but are also electrically addressable by self-aligned electrodes. We have successfully demonstrated controllable coupling barriers of Si_3_N_4_ spacers/c-Si ridges and tunneling barriers of thermally grown SiO_2_, respectively. All Ge QDs within our designer QD arrays are flexible for the configuration design and fabrication of qubits or readout SETs as desired, depending on the c-Si ridges serving as inter-QD coupling barriers or simply acting as reservoirs. Our proposed Ge-QD array approach offers, for the first time, a multi-dimensional parameter space for engineering novel QD electronic devices and optimizing their performance.

## Data Availability

The data presented in this study are available on request from the corresponding author.

## Nomenclature

**Abbreviations**	**Full names**
BG	barrier gate
CMOS	complementary metal-oxide-semiconductor
CB	coupling barrier
DQDs	double quantum dots
EBL	electron-beam lithography
EELS	electron energy loss spectroscopy
EDS	energy dispersive x-ray spectroscopy
Dit	interface trap density
LPCVD	low-pressure chemical vapor deposition
OQDs	octuple QDs
PL	photoluminescence
PG	plunger gate
QQDs	quadruple QDs
qubit	quantum bit
QD	quantum dot
SEM	scanning electron microscopy (SEM)
STEM	scanning transmission electron microscopy
SAED	selected area electron diffraction
SOI	silicon-on-insulator
SET	single-electron transistor
SHT	single-hole transistor
TEM	transmission electron microscopy
TQDs	triple quantum dots
2DEG	two-dimensional electron gas
2DHG	two-dimensional hole gas
VLSI	very large scale integrated circuits
XRD	X-ray diffraction
